# Physical Activity During Pregnancy and Preterm Birth Among Women With Gestational Diabetes

**DOI:** 10.1001/jamanetworkopen.2024.51799

**Published:** 2024-12-19

**Authors:** Wanglong Gou, Congmei Xiao, Xinxiu Liang, Zelei Miao, Meiqi Shi, Yingying Wu, Sha Lu, Xuhong Wang, Yuanqing Fu, Wensheng Hu, Ju-Sheng Zheng

**Affiliations:** 1 Westlake Laboratory of Life Sciences and Biomedicine, Hangzhou, China; 2 Research Center for Industries of the Future, School of Life Sciences, Westlake University, Hangzhou, China; 3 Institute of Basic Medical Sciences, Westlake Institute for Advanced Study, Hangzhou, China; 4 Department of Obstetrics and Gynecology, Hangzhou Women’s Hospital (Hangzhou Maternity and Child Health Care Hospital), Hangzhou, China; 5 Department of Obstetrics, Women’s Hospital, School of Medicine, Zhejiang University, Hangzhou, China; 6 The Affiliated Hangzhou Women’s Hospital of Hangzhou Normal University, Hangzhou, China; 7 School of Medicine, Westlake University, Hangzhou, China

## Abstract

**Importance:**

Physical activity, as a modifiable factor, emerges as a primary intervention strategy for the prevention and management of gestational diabetes (GD). Among women with GD, the association of physical activity during pregnancy with preterm birth remains unclear.

**Objective:**

To examine the association of accelerometer-derived physical activity metrics and patterns with preterm birth among women with GD.

**Design, Setting, and Participants:**

This prospective cohort study recruited pregnant women with GD in Hangzhou, China, from August 2019 to August 2023 as part of the Westlake Precision Birth Cohort study. Statistical analysis was performed between August and November 2023.

**Exposures:**

Wearable accelerometer–derived physical activity metrics and patterns. Measurements of physical activity via wearable accelerometer were performed at a median (IQR) of 25.4 (24.6-26.6) weeks’ gestation.

**Main Outcomes and Measures:**

Preterm birth was determined through the examination of delivery records. Incident preterm birth was defined as the delivery of infants before completing 37 weeks of gestation.

**Results:**

Among the 1427 women meeting the inclusion criteria, the mean (SD) age was 31.3 (3.8) years, and there were 80 cases of preterm birth. An increase in moderate-to-vigorous intensity physical activity (MVPA) and the fraction of physical activity energy expenditure derived from MVPA exhibited an inverse association with preterm birth, with an odds ratio per 30 minutes of 0.64 (95% CI, 0.42-0.98) and an odds ratio per SD of 0.69 (95% CI, 0.55-0.88). In the dose-response analysis, there was a progressive decrease in the odds of preterm birth with increasing duration of MVPA per day, reaching a plateau at approximately 74 minutes per day. Furthermore, the findings indicated that active MVPA (MVPA ≥30 minutes per day), whether it was concentrated into a few days or followed a more regular pattern, had similar beneficial association with preterm birth.

**Conclusions and Relevance:**

In this prospective cohort study, MVPA during pregnancy exhibited an inverse association with preterm birth among women with GD. Concentrated physical activity was associated with similar benefits in reducing preterm birth risk as regular physical activity.

## Key Points

**Question:**

Is physical activity during pregnancy associated with preterm birth among women with gestational diabetes (GD)?

**Findings:**

In this cohort study of 1427 pregnant women with GD, accelerometer-derived moderate-to-vigorous intensity physical activity (MVPA) demonstrated an inverse association with preterm birth. The dose-response curve for MVPA in relation to the risk of preterm birth exhibited an L-shaped pattern, with a steady decline in preterm birth rate up to approximately 74 minutes per day.

**Meaning:**

These findings provide key evidence for the health benefits of MVPA during pregnancy and lay the foundation for establishing physical activity guidelines for pregnant women with GD.

## Teaser

This cohort study examines the association of moderate-to-vigorous intensity physical activity with preterm birth among women in China with gestational diabetes.

## Introduction

Gestational diabetes (GD) is a form of diabetes occurring during pregnancy^1^ that affects approximately 14% of pregnant women globally.^2^ Compared with women with normal glycemia during pregnancy, those with GD have an increased risk of preterm delivery and other pregnancy complications.^3^ Therefore, it is important to establish modifiable interventions aiming at reducing the risk of preterm birth in pregnancies complicated by GD.

Engaging in physical activity is considered to be beneficial for pregnant women in reducing the risk of preterm birth.^4^ Recent guidelines by the World Health Organization have recommended that pregnant women should have at least 150 minutes of moderate-intensity aerobic physical activity per week.^5^ The potential biological mechanisms underlying the association between pregnancy physical activity and the risk of preterm birth are complex and not fully understood. One plausible mechanism is that physical activity may regulate inflammatory responses and oxidative stress, both of which are implicated in the pathogenesis of preterm birth.^6,7^

GD introduces distinct pathophysiological characteristics during pregnancy compared with uncomplicated pregnancies.^8^ Therefore, the results from uncomplicated pregnancies may not be applicable to participants with GD owing to their unique pathophysiological characteristics. It is imperative to conduct specific studies focusing on GD populations to elucidate the association of physical activity with the risk of preterm birth. However, the association of physical activity during pregnancy with preterm birth among individuals with GD remains unclear, and specific exercise guidelines during the pregnancy for individuals with GD are currently lacking.

In this prospective study, we included pregnant women with GD, who were instructed to wear wrist-worn triaxial accelerometers for a duration of 2 weeks. We aimed to examine the associations of the objectively measured physical activity intensity and pattern with the risk of preterm birth in this GD cohort. In addition, we aimed to determine the optimal cutoff of physical activity intensity during pregnancy for these participants.

## Methods

### Study Design and Population

Our study adhered to the Strengthening the Reporting of Observational Studies in Epidemiology (STROBE) reporting guidelines for cohort studies. This cohort study was based on the data from the Westlake Precision Birth Cohort (WeBirth) in Hangzhou, China. All study participants provided written informed consent, and the study was approved by the institutional review board of Westlake University, Hangzhou, China. WeBirth is an ongoing prospective cohort study that recruits pregnant women with hyperglycemia from the Hangzhou Maternity and Child Health Care Hospital in Hangzhou, China, starting from August 2019. Inclusion criteria for WeBirth involved pregnant women (1) who were aged 18 years or older and had received a diagnosis of GD, and with a gestational age ranging from 24 to 28 weeks; and (2) who intended to deliver at Hangzhou Women’s Hospital and remain in Hangzhou with their children for 4 or more years. Pregnant women with cancers or infectious diseases, including those testing positive for the 3 major markers of hepatitis B (hepatitis B surface antigen, hepatitis B core antigen, and hepatitis B e antigen), syphilis antibodies, or HIV infection were excluded from the study. GD diagnosis was based on the International Association of Diabetes and Pregnancy Study Groups criteria, which encompasses fasting plasma glucose greater than or equal to 91.9 mg/dL (to convert to millimoles per liter, multiply by 0.0555), and/or 1 hour plasma glucose greater than or equal to 180.0 mg/dL, and/or 2-hour plasma glucose greater than or equal to 153.3 mg/dL.^9^

Upon enrollment, participants were required to complete several interviewer-administered questionnaires, providing information on demographics, dietary and lifestyle factors, medical histories, and health status before and during pregnancy. In addition, participants were instructed to wear a triaxial accelerometer (AX3; Axivity) on their dominant wrist continuously for a period of 14 consecutive days. The median (IQR) interval between GD diagnosis and recruitment into the present study was 0 (0-6) days. Further details about the design and methods of the WeBirth study can be found elsewhere.^10^

Up to August 2023, a total of 1902 participants with GD were recruited in the WeBirth study. Participants were excluded if they lacked follow-up information (159 participants), did not have valid wearable device-measured physical activity data (208 participants), or if information on covariates was unavailable (108 participants) (eFigure 1 in Supplement 1).

### Assessments of Preterm Birth and Physical Activity

Preterm birth, as defined by the World Health Organization, refers to the delivery of infants before completing 37 weeks of gestation.^11^ Indications for maternal admission and delivery were identified from the hospital admission and delivery records.

Physical activity was assessed using a wrist-worn triaxial accelerometer, and the data were analyzed using the same pipeline as in previous studies.^12,13,14^ The raw acceleration data were collected at a resolution of 100 Hz. To ensure accuracy, the acceleration data were calibrated to local gravity, and ambient temperature was taken into account during the calibration process.^15^ The Euclidean norm of calibrated acceleration in the 3 axes was calculated after removing machine noise using a fourth-order Butterworth low-pass filter at 20 Hz. From this, 1*g* was subtracted, and any negative values were truncated to zero.^12^ Nonwear time was considered to be time periods of 60 minutes or more where the SD of acceleration in each of the 3 axes was less than 13*g*.^16^ For participants who had wearable devices but had periods of nonwear time, we imputed these missing data using the mean of similar time-of-day segments from that individual, as in previous studies.^12,13,14^

We estimated instantaneous physical activity energy expenditure (PAEE) from wrist movement intensity using the previously established pipeline.^14^ We calculated the mean daily PAEE (kilojoules per kilograms^–1^ per day^–1^) to represent the total physical activity volume during the wearing time.^14,17^ Moderate-to-vigorous intensity physical activity (MVPA) was calculated as the energy expenditure from physical activity time above 3 metabolic equivalents of task. From a practical perspective, we provided a list of activities with a metabolic equivalents of task value of 3 in the eTable 1 in Supplement 1, referring to the Compendium of Physical Activities.^18^ The fraction of PAEE from MVPA (FMVPA) was defined as MVPA divided by total PAEE, expressed as a percentage.^14,17^

### Statistical Analysis

Statistical analysis was performed between August and November 2023. We performed all the statistical analyses in Stata statistical software version 16 (StataCorp) or R statistical software version 4.1 (R Project for Statistical Computing). We used logistic regression models to evaluate the associations of the accelerometer-derived physical activity metrics (PAEE, MVPA, and FMVPA) with preterm delivery. Model 0 is an unadjusted model. Model 1 includes adjustments for age, gestational week at baseline, prepregnancy body mass index, and PAEE (only for MVPA). Model 2 is the same as model 1, plus household income, educational level, smoking, alcohol drinking, and parity. Two-tailed *P* < .05 was considered statistically significant. In addition, we evaluated the associations of preeclampsia with preterm birth, as well as the association of MVPA with preeclampsia, on the basis of model 2. Furthermore, we examined the interaction of age and prepregnancy body mass index with physical activity metrics on preterm birth by adding a cross-product term into the aforementioned logistic regression model. Analyses within subgroups defined by the variables were performed if the *P* value for interaction was less than .05.

Several sensitivity analyses based on model 2 were conducted to test the robustness of results. These included (1) including the accelerometer wear time as an additional covariate, (2) including the recruited period (before or after the COVID-19 pandemic) as an additional covariate into the model to assess the potential influence of lifestyle changes during the COVID-19 pandemic,^19^ (3) excluding those with accelerometer wear time less than 5 days (24 participants), (4) excluding participants who were advised by their doctors to undergo bed rest to maintain their pregnancy to reduce the possibility of reverse causation bias (16 participants), (5) excluding the twin and other multiples pregnant participants (16 participants), (6) including history of preterm birth as additional covariate, (7) including preeclampsia as additional covariate, (8) including medication for the treatment of GD as additional covariate, (9) including occupation as an additional covariate, and (10) treating gestational age at delivery as a continuous outcome.

We conducted several secondary analyses to further explore the association between physical activity and preterm birth. First, we conducted separate analyses for 2 clinical subtypes of preterm birth: spontaneous and medically indicated preterm birth.^20^ Second, we divided preterm birth into subcategories on the basis of gestational age at the time of birth: very preterm (28 to <32 weeks) and moderate to late preterm (32 to 37 weeks), repeating our analysis for each category. Finally, we examined the association of physical activity with preterm birth separately among primiparous and nonprimiparous participants.

To provide actionable guidance for physical activity, we then investigated the dose-response association of MVPA with preterm birth with 2 approaches using model 2: (1) comparing progressively higher 30-minute per day categories against a reference group with less than 30 minutes per day, and (2) utilization of restricted cubic spline terms (4 knots) to evaluate the dose-response association between MVPA and preterm delivery, with MVPA equal to 30 minutes per day serving as the reference.

We next used model 2 to examine the association of physical activity pattern with preterm delivery. To achieve this, we first categorized participants into 2 groups: physically inactive (MVPA <30 minutes per day) or physically active (MVPA ≥30 minutes per day). Within the active group, we further subcategorized individuals according to the intensity and variation of MVPA during the period of wearing accelerometer. Specifically, we calculated the coefficient of variation of MVPA for each individual and identified individuals with lower MVPA variation (coefficient of variation below the 25th percentile) as the regularly active group. Participants with a similar MVPA intensity compared with the regularly active group (matched in a 1:1 ratio according to MVPA), but with higher MVPA variation (coefficient of variation above the 25th percentile), were defined as the weekend warrior group (ie, those who engaged in concentrated patterns of physical activity).^21^

## Results

### Population Characteristics

The study encompassed a population of 1427 participants, with a mean (SD) age of 31.3 (3.8) years at recruitment. The accelerometers were worn at median (IQR) of 25.4 (24.6-26.6) weeks’ gestation. Among the study cohort, 80 cases of preterm delivery were identified, constituting 5.6% of the participants. Participants wore the wearable device for an mean (SD) of 11.4 (2.3) days. There were 21 cases of medically indicated preterm delivery, 6 of which were due to preeclampsia (eTable 2 in Supplement 1). Baseline characteristics of the study participants stratified by preterm birth are shown in Table 1; characteristics stratified by MVPA are presented in eTable 3 in Supplement 1. Preeclampsia was associated with preterm birth (odds ratio [OR], 3.78; 95% CI, 1.46-9.78) but not with physical activity (OR, 0.75; 95% CI, 0.55-1.04; *P* = .08). There were no demographic differences between the participants included and those excluded in our study (eTable 4 in Supplement 1).

**Table 1.** Descriptive Characteristics of Participants by Preterm Birth

Characteristic	Participants, No. (%)		
	Total (N = 1427)	No preterm birth (n = 1347)	Preterm birth (n = 80)
Moderate-to-vigorous intensity physical activity, mean (SD), min/d	84.0 (38.1)	84.5 (38.0)	75.5 (40.2)
Fraction of physical activity energy expenditure accumulated from moderate-to-vigorous intensity physical activity, mean (SD), %	33.0 (10.2)	33.2 (10.1)	29.7 (11.2)
Physical activity energy expenditure, mean (SD), kJ kg^–1^ d^–1^	43.8 (10.9)	43.9 (10.8)	42.3 (12.3)
Accelerometer wear time, mean (SD), d	11.4 (2.3)	11.4 (2.3)	11.3 (2.8)
Age, mean (SD), y	31.3 (3.8)	31.2 (3.8)	32.0 (4.1)
Gestational age at baseline, mean (SD), wk	25.9 (1.9)	25.9 (1.9)	25.8 (2.3)
Prepregnancy body mass index, mean (SD)^a^	22.3 (3.6)	22.2 (3.6)	22.5 (3.8)
Primiparity	911 (63.8)	857 (63.6)	54 (67.5)
Pregnant with twins	16 (1.1)	7 (0.5)	9 (11.3)
Pregnant with preeclampsia	36 (2.5)	30 (2.2)	6 (7.5)
Pregnant with medication treatment	15 (1.1)^b^	12 (0.9)	3 (3.8)
Pregnant with history of preterm birth	18 (1.3)	13 (0.97)	5 (6.3)
Education			
High school or vocational school	164 (11.5)	155 (11.5)	9 (11.3)
University or professional school	1053 (73.8)	998 (74.1)	55 (68.8)
Greater than university	210 (14.7)	194 (14.4)	16 (20.0)
Annual household income, ¥^c^			
<100 000	339 (23.8)	317 (23.5)	22 (27.5)
100 000-200 000	552 (38.7)	529 (39.3)	23 (28.7)
>200 000	536 (37.6)	501 (37.2)	35 (43.8)
Current smoking	50 (3.5)	45 (3.3)	5 (6.3)
Current drinking	43 (3.0)	41 (3.0)	2 (2.5)
Recruitment after COVID-19			
No	224 (15.7)	211 (94.2)	13 (5.8)
Yes	1203 (84.3)	1136 (94.4)	67 (5.6)

^a^ Body mass index is calculated as weight in kilograms divided by height in meters squared.

^b^ Among the included participants, 11 were treated with metformin and 4 were treated with insulin.

^c^ As of November 8, 2024, ¥1.00 = US $0.14.

### Association of Physical Activity With Preterm Birth

We observed an inverse association of physical activity during pregnancy with preterm delivery (Table 2). The OR for preterm birth was 0.82 (95% CI, 0.68-0.99) for MVPA (per 30 minutes) and 0.70 (95% CI, 0.56-0.89) for the FMVPA (per SD unit) (Table 2). These findings remained consistent after further adjustment for the potential confounders, with ORs of 0.64 (95% CI, 0.42-0.98) for MVPA and 0.69 (95% CI, 0.55-0.88) for FMVPA in model 2 (Table 2). Notably, we did not observe a significant association between PAEE and preterm delivery.

**Table 2.** Associations of Physical Activity With Preterm Birth^a^

Physical activity metrics	OR (95% CI)		
	Model 0	Model 1	Model 2
MVPA, min	0.82 (0.68-0.99)	0.66 (0.43-0.99)	0.64 (0.42-0.98)
FMVPA, %	0.70 (0.56-0.89)	0.70 (0.55-0.89)	0.69 (0.55-0.88)
PAEE, kJ kg^–1^ d^–1^	0.86 (0.69-1.09)	0.85 (0.67-1.07)	0.86 (0.68-1.09)

Abbreviations: FMVPA, fraction of physical activity energy expenditure accumulated from moderate-to-vigorous-intensity physical activity; MVPA, moderate-to-vigorous-intensity physical activity; OR, odds ratio; PAEE, physical activity energy expenditure.

^a^ Logistic regression was used to estimate the ORs and 95% CI for preterm birth per 30-minute increase in MVPA and per SD change in FMVPA and PAEE. A total of 1427 participants were included in the analysis, with a total of 80 cases of preterm delivery identified. Model 0 is an unadjusted model. Model 1 includes adjustments for age, gestational week at baseline, prepregnancy body mass index and PAEE (only for MVPA). Model 2 is the same as model 1 plus household income, educational level, smoking, alcohol drinking, and parity.

We found a significant interaction between age and the FMVPA in relation to preterm delivery (*P* for interaction = .047). The inverse association between the FMVPA and preterm birth was slightly more pronounced among older participants (aged >31 years) (eTable 5 in Supplement 1).

**Figure 1.**
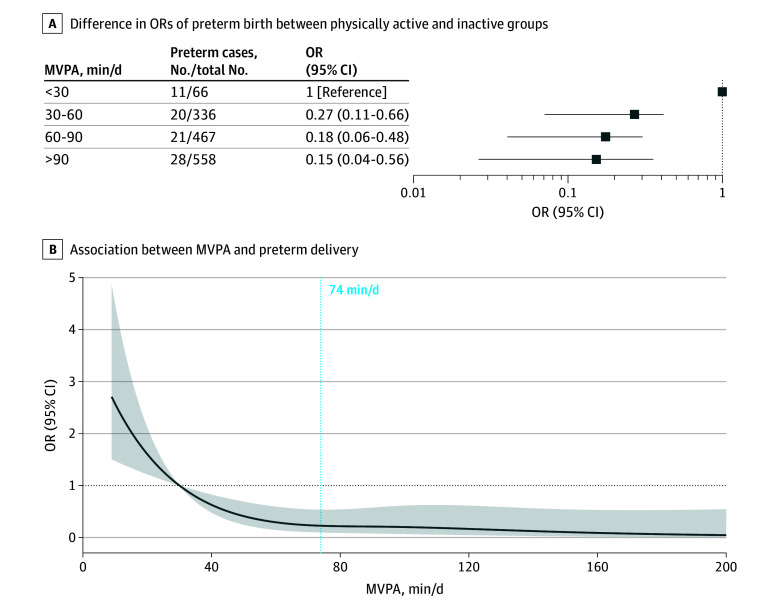
Dose-Response Association of Moderate-to-Vigorous-Intensity Physical Activity (MVPA) With Preterm Birth A, Logistic regression was used to estimate the difference in odds ratios (ORs) of preterm birth between the physically active groups (MVPA ≥30 min/d) and inactive group (MVPA <30 min/d). The model was adjusted for physical activity energy expenditure, age, gestational week at baseline, prepregnancy body mass index, household income, educational level, smoking, alcohol drinking, and parity. B, Restricted cubic spline analysis was used to assess the shape of the association between MVPA and preterm delivery, adjusting for the same set of confounders as the aforementioned logistic regression. Here, MVPA equal to 30 minutes per day served as the reference. The y-axis represents the OR of preterm birth, with the shaded area indicating the corresponding 95% CIs.

The results of various sensitivity analyses led to similar conclusions as the aforementioned main results (eTable 6 in Supplement 1). In addition, our secondary analyses, including those examining different subtypes of preterm birth and categorizations based on parity, did not substantially alter the findings (eTables 7 and 8 in Supplement 1). Compared with the MVPA less than 30 group, the OR for the 30 to 60 MVPA group was 0.27 (95% CI, 0.11-0.66), that for the 60 to 90 MVPA group was 0.18 (95% CI, 0.06-0.48), and that for the greater than 90 MVPA group was 0.15 (95% CI, 0.04-0.56) (Figure 1A). The dose-response curve for MVPA in association with the risk of preterm birth exhibited an L-shaped pattern, showing a steady decline in preterm birth rate with an increase in MVPA up to approximately 74 minutes per day (Figure 1B).

### Association of Physical Activity Pattern (Weekend Warrior and Regularly Active) With Preterm Birth

**Figure 2.**
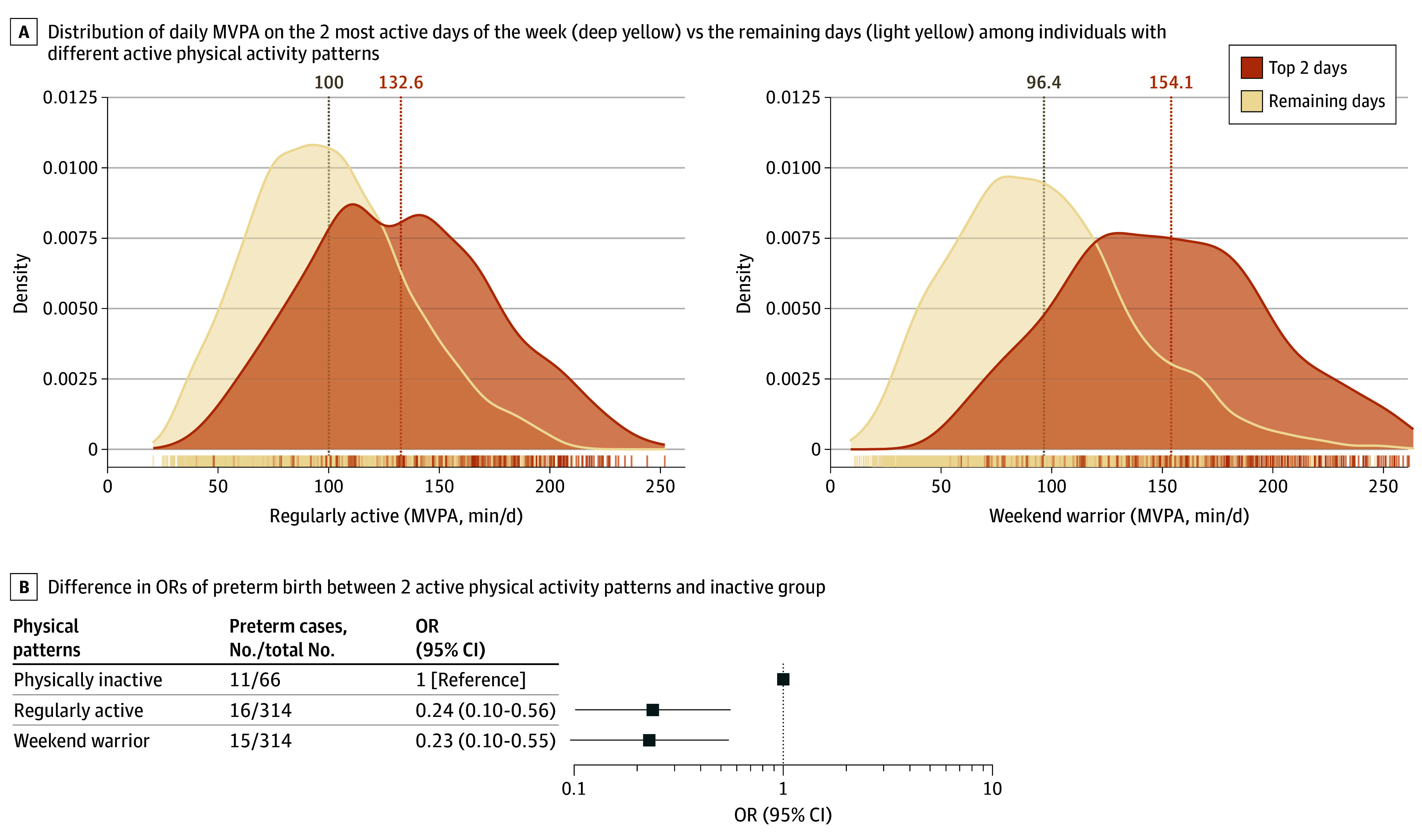
Association of Physical Activity Patterns With Preterm Birth A, Distribution of daily moderate-to-vigorous-intensity physical activity (MVPA) on the 2 most active days of the week vs the remaining days among individuals with different active physical activity patterns. B, Association of physical activity patterns with preterm birth. Logistic regression was used to estimate the difference in odds ratios (ORs) of preterm birth between 2 active physical activity patterns and inactive group (MVPA <30 min/d), adjusting for age, gestational week at baseline, prepregnancy body mass index, physical activity energy expenditure, household income, educational level, smoking, alcohol drinking, and parity.

In total, 1361 participants were categorized into the physically active group (MVPA ≥30 minutes per day). In addition, 66 participants were classified as physically inactive group (MVPA <30 minutes per day). Within the physically active group, 314 were classified as the regularly active group and 314 as the weekend warrior group. Participants in the weekend warrior group exhibited a similar MVPA intensity as the regularly active group (mean [SD], 104.6 [34.2] minutes vs 104.1 [32.6] minutes) but with higher MVPA variation (eFigure 2 in Supplement 1). As demonstrated in the Figure 2A, participants in the weekend warrior group had substantially more MVPA on their 2 most active days vs the remaining days, whereas those in the regularly active group had more consistent MVPA. Both active groups were associated with lower odds of preterm birth (Figure 2B). Compared with physically inactive participants, multivariable adjusted ORs for preterm birth were 0.24 (95% CI, 0.10-0.56) for regularly active participants and 0.23 (95% CI, 0.10-0.55) for weekend warrior participants.

## Discussion

In this prospective cohort study, we found that physical activity during pregnancy exhibited an inverse association with preterm birth among women with GD. We observed an L-shaped association between the MVPA and preterm birth risk, with a steady decline in preterm birth rate with an increase in MVPA up to approximately 74 minutes per day. Compared with being physically inactive, both weekend warrior and regularly active patterns of physical activity demonstrated a similar protective association with preterm birth.

We used a wearable accelerometer to assess the intensity and patterns of physical activity among more than 1400 participants with GD during pregnancy. The overall estimated activity energy expenditure from the accelerometer has been demonstrated with a higher agreement with the reference-standard doubly labeled water method in UK adults.^22^ Our findings revealed that although the overall association of MVPA with preterm birth, assuming linearity, is significant, closer examination reveals that the association is not linear. Furthermore, maternal age is an independent risk factor for preterm birth, and the risk of preterm birth typically increases as maternal age surpasses 30 years.^23^ Our findings emphasize the potential protective role of MVPA in mitigating the risk of preterm birth, with a trend toward greater benefits in older women.

In China, there are no specific physical activity guidelines for pregnant women.^24^ In Denmark, pregnant women are recommended to have at least 30 minutes of (unspecified) daily, moderate-intensity physical exercise.^25^ Our study findings support this recommendation for women with GD, showing a steady decline in the risk of preterm birth for participants with more than 30 minutes per day. In addition, our study highlights that achieving more than 74 minutes of MVPA per day may be sufficient to maximize the beneficial effects of MVPA in the prevention of preterm delivery. These results provide valuable support for the development of guidelines and recommendations regarding physical activity for women with GD, potentially helping to prevent adverse pregnancy outcomes in this population.

The term *weekend warrior* describes a physical activity pattern in which the majority of MVPA is concentrated and distributed within a few days. Several studies^21,26^ have suggested that both weekend warrior and regularly active physical activity patterns provide similar cardiovascular and mortality benefits. However, to our knowledge, there are no prospective studies that have examined whether the weekend warrior pattern has comparable benefits in terms of preterm birth risk vs regularly active physical activity. Our analysis revealed that the weekend warrior pattern is associated with similar reductions in preterm birth risk as the regularly active pattern. This finding is especially pertinent for individuals who have time constraints, as it suggests that engaging in shorter time frame of physical activity can still yield positive results in terms of preventing preterm delivery.

This study has several strengths. First, to our knowledge, this is the largest cohort study examining the association of objectively measured physical activity with preterm birth in pregnant women. The results highlight the importance of physical activity during pregnant for GD populations and provide recommendations of optimal MVPA for these participants. Second, we provide novel comparisons between weekend warrior, regularly active, and physically inactive participants based on the frequency and intensity of physical activity for the preterm outcome, which, to our knowledge, has never been conducted in pregnant women previously.

### Limitations

There are several limitations that should be considered when interpreting the results of the present study. First, wearing the accelerometer may induce social desirability and incentivize the participants to enhance the physical activity, although it is still not clear about the potential influence of this change; moreover, it remains uncertain whether a 14-day accelerometer measurement during this period accurately reflects longer-term physical activity patterns. Therefore, future research should confirm these findings in other populations by using repeated measured physical activity. Second, it is important to note that although we include a large number of participants with GD, the higher socioeconomic status of our cohort may have contributed to the lower incidence of preterm birth. Third, factors such as diet and lifestyle variables and preconception physical activity levels were not thoroughly examined, which could potentially introduce bias into the estimated effects. Fourth, our study was conducted among pregnant individuals with GD, and the relatively active nature of our cohort participants may introduce selection bias, which may influence the effect estimates. External generalizability of the study findings remains to be tested. Fifth, the causal relationship between physical activity and preterm birth remains uncertain owing to the observational nature of the studies. It is unclear whether there is an independent association between these 2 variables, or whether the observed association can be attributed to intermediate factors such as reduced risk of preeclampsia.

## Conclusions

In this prospective cohort study, we found that MVPA during pregnancy was associated with a lower risk of preterm birth among women with GD. Most interestingly, concentrated physical activity provided similar benefits in reducing preterm birth risk as regular physical activity. Our study provides key evidence for health benefit of MVPA during pregnancy and lays the foundation for the establishment of physical activity guidelines for pregnant women with GD.

## Notes

Supplement 1. **eTable 1.** A list of activities with a MET value of 3 **eTable 2.** Reasons for medically indicated preterm births **eTable 3.** Comparison of baseline characteristics between participants with higher and lower moderate-to-vigorous-intensity physical activity in our study **eTable 4.** Comparison of baseline characteristics between participants included and those excluded in our study **eTable 5.** Association of the fraction of moderate-to-vigorous intensity physical activity from the physical activity energy expenditure with preterm birth stratified by the median age of participants **eTable 6.** Sensitivity analyses of associations between physical activity and preterm birth **eTable 7.** Secondary analyses of associations between physical activity and preterm birth **eTable 8.** Association of physical activity with preterm birth stratified by primiparity **eFigure 1.** Flowchart of study sample selection **eFigure 2.** Distribution of daily MVPA and its coefficient of variation among individuals with different active physical activity patterns

Supplement 2.
Data Sharing Statement
