# Supplementary material for: Physical Activity During Pregnancy and Preterm Birth Among Women With Gestational Diabetes
Source: JAMA Netw Open. 2024 Dec 19;7(12):e2451799. doi: 10.1001/jamanetworkopen.2024.51799 (PMC11659911; doi:10.1001/jamanetworkopen.2024.51799)
Supplement: Supplement 1. — eTable 1. A list of activities with a MET value of 3 eTable 2. Reasons for medically indicated preterm births eTable 3. Comparison of baseline characteristics between participants with higher and lower moderate-to-vigorous-intensity physical activity in our study eTable 4. Comparison of baseline characteristics between participants included and those excluded in our study eTable 5. Association of the fraction of moderate-to-vigorous intensity physical activity from the physical activity energy expenditure with preterm birth stratified by the median age of participants eTable 6. Sensitivity analyses of associations between physical activity and preterm birth eTable 7. Secondary analyses of associations between physical activity and preterm birth eTable 8. Association of physical activity with preterm birth stratified by primiparity eFigure 1. Flowchart of study sample selection eFigure 2. Distribution of daily MVPA and its coefficient of variation among individuals with different active physical activity patterns [file jamanetwopen-e2451799-s001.pdf]

Supplemental Online Content

Gou W, Xiao C, Liang X, et al. Physical activity during pregnancy and preterm birth among women with gestational diabetes. *JAMA Netw Open*. 2024;7(12):e2451799. doi:10.1001/jamanetworkopen.2024.51799

eTable 1. A list of activities with a MET value of 3
eTable 2. Reasons for medically indicated preterm births
eTable 3. Comparison of baseline characteristics between participants with higher and lower moderate-to-vigorous-intensity physical activity in our study
eTable 4. Comparison of baseline characteristics between participants included and those excluded in our study
eTable 5. Association of the fraction of moderate-to-vigorous intensity physical activity from the physical activity energy expenditure with preterm birth stratified by the median age of participants
eTable 6. Sensitivity analyses of associations between physical activity and preterm birth
eTable 7. Secondary analyses of associations between physical activity and preterm birth
eTable 8. Association of physical activity with preterm birth stratified by primiparity
eFigure 1. Flowchart of study sample selection
eFigure 2. Distribution of daily MVPA and its coefficient of variation among individuals with different active physical activity patterns

This supplemental material has been provided by the authors to give readers additional information about their work.

© 2024 Gou W et al. *JAMA Network Open.*

eTable 1. A list of activities with a MET value of 3.

**Walking**
Walking, 2.5 mph, firm, level surface
Walking, treadmill, 2.0 to 2.4 mph (3.2 to 3.9 km/h), 0% grade
**Home activity**
Vacuuming, general, moderate effort
Making bed, changing linens
Sweeping garage, sidewalk, or outside house
Implied walking, putting away household items, moderate effort
Walking and carrying small child, child weighing 15 lbs or more
Elder care, disabled adult, bathing, dressing, moving into and out of bed, only active periods
**Lawn and garden**
Walking, gathering gardening tools
Walking, applying fertilizer or seeding a lawn, push applicator
Implied walking/standing – picking up yard, light, picking flowers or vegetables
Walking, gathering gardening tools
**Conditioning exercises**
Body weight resistance exercises (e.g., squat, lunge, push-up, crunch), general
Yoga, Hot
**Dancing**: waltz, foxtrot, slow dancing, samba tango, rumba, 19th century dance, mambo, cha cha

eTable 2. Reasons for medically indicated preterm births.

Patient ID	Conditions
1	In vitro fertilization baby, hypertension, abnormal fetal position, thrombophilia
2	Preeclampsia
3	Preeclampsia
4	Premature rupture of membranes, fetal distress
5	Cholestasis
6	Cord around the body and fetal arrhythmia
7	Abnormal fetal position
8	Other maternal systemic diseases
9	Fetal distress, other maternal systemic diseases
10	Fetal distress
11	Cholestasis, in vitro fertilization baby
12	Cholestasis
13	Preeclampsia
14	Cholestasis
15	Other maternal systemic diseases, in vitro fertilization baby
16	Preeclampsia
17	Abnormal fetal position, other maternal systemic diseases
18	Premature rupture of membranes, other maternal systemic diseases
19	Preeclampsia
20	Other maternal systemic diseases
21	Preeclampsia

© 2024 Gou W et al. *JAMA Network Open.*

eTable 3. Comparison of baseline characteristics between participants with higher and lower moderate-to-vigorous-intensity physical activity in our study.

	Patients, No (%)	
	MVPA< 78.6 min / day [a]	MVPA≥ 78.6 min / day
Characteristic	n=714	n=713
Age, mean (SD), years	31.23 (3.89)	31.28 (3.80)
Gestational week at baseline, mean (SD), weeks	25.98 (2.01)	25.75 (1.74)
Pre-pregnancy BMI, mean (SD), kg/m^2	22.26 (3.43)	22.24 (3.70)
Primiparity	458 (64.1%)	453 (63.5%)
Pregnant with preeclampsia	22 (3.1%)	14 (2.0%)
Pregnant with medication treatment	6 (0.4%)	9 (0.8%)
Pregnant with history of preterm birth	10 (1.4%)	8 (1.1%)
Education		
≤ High school or vocational school	73 (10.2%)	92 (12.9%)
University or professional school	546 (76.5%)	506 (71.0%)
> University	95 (13.3%)	115 (16.1%)
Household income, ¥/year		
< 100,000	164 (23.0%)	175 (24.5%)
100,000 – 200,000	273 (38.2%)	280 (39.3%)
> 200,000	277 (38.8%)	258 (36.2%)
Current smoking	30 (4.2%)	21 (2.9%)
Current drinking	22 (3.1%)	21 (2.9%)
After COVID recruitment		
No	97 (13.6%)	127 (17.8%)
Yes	617 (86.4%)	586 (82.2%)

Abbreviations: MVPA, Moderate-to-Vigorous Intensity Physical Activity.

[a] 78.6 is the median value of MVPA.

© 2024 Gou W et al. *JAMA Network Open.*

eTable 4. Comparison of baseline characteristics between participants included and those excluded in our study.

	Patients, No (%)	
	Included	Excluded
Characteristic	n=1,427	n=475
Age, mean (SD), years	31.3 (3.8)	30.82 (3.72)
Gestational week at baseline, mean (SD), weeks	25.9 (1.89)	26.03 (1.96)
Pre-pregnancy BMI, mean (SD), kg/m^2	22.3 (3.6)	21.92 (3.65)
Primiparity	911 (63.8%)	331 (69.7%)
Pregnant with preeclampsia	36 (2.5%)	12 (2.5%)
Pregnant with medication treatment	15 (1.1 %)	2 (0.42%)
Pregnant with history of preterm birth	18 (1.3%)	0 (0)
Education		
≤ High school or vocational school	164 (11.5%)	58 (13.6%)
University or professional school	1053 (73.8%)	318 (74.5%)
> University	210 (14.7%)	51 (11.9%)
Household income, ¥/year		
< 100,000	339 (23.8%)	63 (19.7%)
100,000 – 200,000	552 (38.7%)	122 (38.2%)
> 200,000	536 (37.6%)	134 (42.0%)
Current smoking	50 (3.5%)	28 (6.6%)
Current drinking	43 (3.0%)	10 (2.3%)
After COVID recruitment		
No	224 (15.7%)	101 (21.3%)
Yes	1,203 (84.3%)	374 (78.7%)

© 2024 Gou W et al. *JAMA Network Open.*

eTable 5. Association of the fraction of moderate-to-vigorous intensity physical activity from the physical activity energy expenditure with preterm birth stratified by the median age of participants.

Age	No. of preterm cases / total No.	OR (95% CI)	$P_{interaction}$=0.047
<31 years	31 / 714	0.78 (0.53 - 1.16)	
≥31years	49 / 713	0.68 (0.50 - 0.91)	

Logistic regression was used to estimate the odds rations and 95% CI for preterm birth per standard deviation change in the fraction of moderate-to-vigorous intensity physical activity from the physical activity energy expenditure, adjusting for age, gestational week at baseline, pre-pregnancy BMI, household income, educational level, smoking, alcohol drinking and parity.

© 2024 Gou W et al. *JAMA Network Open.*

eTable 6. Sensitivity analyses of associations between physical activity and preterm birth.

Model	No. of preterm cases / total No.	MVPA, min / day	FMVPA, %	PAEE, kJ kg–1 day–1
OR, multivariable-adjusted [a]	80 / 1427	0.64 (0.42 - 0.98)	0.69 (0.55 - 0.88)	0.86 (0.68 - 1.09)
OR, multivariable-adjusted, including wear time as additional covariate	80 / 1427	0.64 (0.42 - 0.98)	0.69 (0.55 - 0.88)	0.86 (0.68 - 1.10)
OR, multivariable-adjusted, including recruited period as additional covariate	80 / 1427	0.65 (0.42 - 0.98)	0.69 (0.55 - 0.88)	0.85 (0.67 - 1.08)
OR, multivariable-adjusted, excluding participants with accelerometer wear time less than 5 days	80 / 1403	0.64 (0.41 - 0.98)	0.70 (0.55 - 0.89)	0.87 (0.69 - 1.10)
OR, multivariable-adjusted, excluding participants who were advised by their doctors to undergo bed rest to maintain their pregnancy	79 / 1411	0.62 (0.40 - 0.95)	0.70 (0.55 - 0.89)	0.87 (0.69 - 1.10)
OR, multivariable-adjusted, excluding the twins and other multiples pregnant participants	71 / 1411	0.61 (0.39 - 0.95)	0.77 (0.60 - 0.98)	0.96 (0.74 - 1.23)
OR, multivariable-adjusted, including history of preterm birth as additional covariate	80 / 1427	0.65 (0.42 - 0.99)	0.71 (0.56 - 0.89)	0.86 (0.68 - 1.09)
OR, including preeclampsia as additional covariate	80 / 1427	0.65 (0.43 - 0.99)	0.71 (0.56 - 0.90)	0.87 (0.69 - 1.10)
OR, including medication for the treatment of GDM as additional covariate	80 / 1427	0.65 (0.43 - 0.99)	0.70 (0.55 - 0.88)	0.86 (0.68 - 1.09)
OR, including occupation as additional covariate	80 / 1427	0.62 (0.41 - 0.96)	0.70 (0.55 - 0.89)	0.88 (0.69 - 1.11)
Beta, gestational age at delivery as continuous outcome [b]	80 / 1427	0.22 (0.09 - 0.36)	0.17 (0.096 - 0.25)	0.088 (0.0084 - 0.17)

[a] Logistic regression was used to estimate the odds ratios and 95% CI for preterm birth for preterm birth per 30-minute increase in MVPA and per standard deviation change in FMVPA and PAEE. Covariates included in the multivariable-adjusted model were age, gestational week at baseline, pre-pregnancy BMI, PAEE (only for MVPA) household income, educational level, smoking, alcohol drinking and parity. PAEE: physical activity energy expenditure; MVPA: Moderate-to-Vigorous Intensity Physical Activity; FMVPA: fraction of PAEE from MVPA.

[b] Linear regression was used to estimate the beta and 95% CI for gestational age at delivery per 30-minute increase in MVPA and per standard deviation change in FMVPA and PAEE. The adjusted covariates were the same as those used in the logistic regression.

© 2024 Gou W et al. *JAMA Network Open.*

eTable 7. Secondary analyses of associations between physical activity and preterm birth.

	No. of preterm cases / total No.	MVPA, min / day	FMVPA, %	PAEE, kJ kg–1 day–1
Spontaneous preterm	59 / 1406			
OR, preterm birth as outcome		0.60 (0.36 - 0.98)	0.73 (0.55 - 0.96)	0.87 (0.66 - 1.15)
Beta, gestational age at delivery as outcome		0.21 (0.079 - 0.34)	0.15 (0.073 - 0.22)	0.076 (-0.0012 - 0.15)
Medically indicated preterm	21 / 1367			
OR, preterm birth as outcome		0.78 (0.36 - 1.70)	0.61 (0.39 - 0.96)	0.81 (0.52 - 1.28)
Beta, gestational age at delivery as outcome		0.099 (-0.0019 - 0.20)	0.09 (0.03 - 0.15)	0.05 (-0.02 - 0.11)
Moderate to late preterm	74 / 1421			
OR, preterm birth as outcome		0.71 (0.46 - 1.09)	0.74 (0.58 - 0.94)	0.88 (0.69 - 1.12)
Beta, gestational age at delivery as outcome		0.17 (0.047 - 0.30)	0.14 (0.067 - 0.21)	0.073 (-0.00038 - 0.15)
Very preterm	6 / 1352			
OR, preterm birth as outcome		0.17 (0.029 - 0.98)	0.37 (0.16 - 0.87)	0.62 (0.27 - 1.44)
Beta, gestational age at delivery as outcome		0.14 (0.029 - 0.25)	0.098 (0.036 - 0.16)	0.054 (-0.01 - 0.12)

Logistic regression was used to estimate the odds ratios and 95% CI for preterm birth per30-minute increase in MVPA and per standard deviation change in FMVPA and PAEE. Linear regression was employed to estimate the beta coefficients and corresponding 95% CI for gestational week at delivery. Covariates included in the model were age, gestational week at baseline, pre-pregnancy BMI, PAEE (only for MVPA) household income, educational level, smoking, alcohol drinking and parity. PAEE: physical activity energy expenditure; MVPA: Moderate-to-Vigorous Intensity Physical Activity; FMVPA: fraction of PAEE from MVPA.

© 2024 Gou W et al. *JAMA Network Open.*

eTable 8. Association of physical activity with preterm birth stratified by primiparity.

Primiparity	No. of preterm cases / total No.	MVPA, min / day	FMVPA, %	PAEE, kJ kg^{-1} day^{-1}
Yes				
OR, preterm birth as outcome	54 / 911	0.68 (0.40 - 1.17)	0.67 (0.51 - 0.89)	0.78 (0.58 - 1.04)
Beta, gestational age as outcome	54 / 911	0.22 (0.039 - 0.40)	0.19 (0.1 - 0.29)	0.13 (0.02 - 0.23)
No				
OR, preterm birth as outcome	26 / 516	0.53 (0.26 - 1.07)	0.75 (0.50 - 1.14)	0.97 (0.66 - 1.44)
Beta, gestational age as outcome	26 / 516	0.27 (0.066 - 0.46)	0.14 (0.015 - 0.26)	0.019 (-0.1 - 0.14)

Logistic regression was used to estimate the odds ratios and 95% CI for preterm birth per 30-minute increase in MVPA and per standard deviation change in FMVPA and PAEE. Linear regression was employed to estimate the beta coefficients and corresponding 95% CI for gestational week at delivery. Covariates included in the model were age, gestational week at baseline, pre-pregnancy BMI, PAEE (only for MVPA) household income, educational level, smoking, alcohol drinking and parity. PAEE: physical activity energy expenditure; MVPA: Moderate-to-Vigorous Intensity Physical Activity; FMVPA: fraction of PAEE from MVPA.

© 2024 Gou W et al. *JAMA Network Open.*

eFigure 1. Flowchart of study sample selection. Abbreviations: WeBirth, Westlake Precision Birth Cohort.

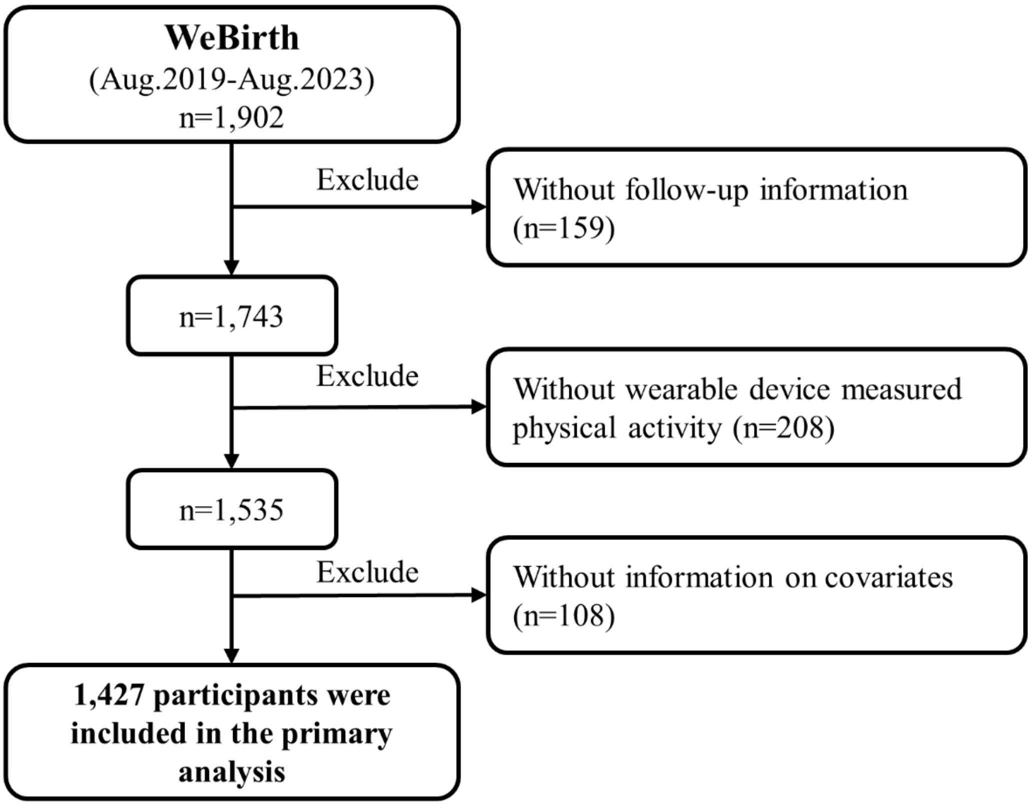

© 2024 Gou W et al. *JAMA Network Open.*

eFigure 2. Distribution of daily MVPA and its coefficient of variation among individuals with different active physical activity patterns. Abbreviations: MVPA, moderate-to-vigorous-intensity physical activity.

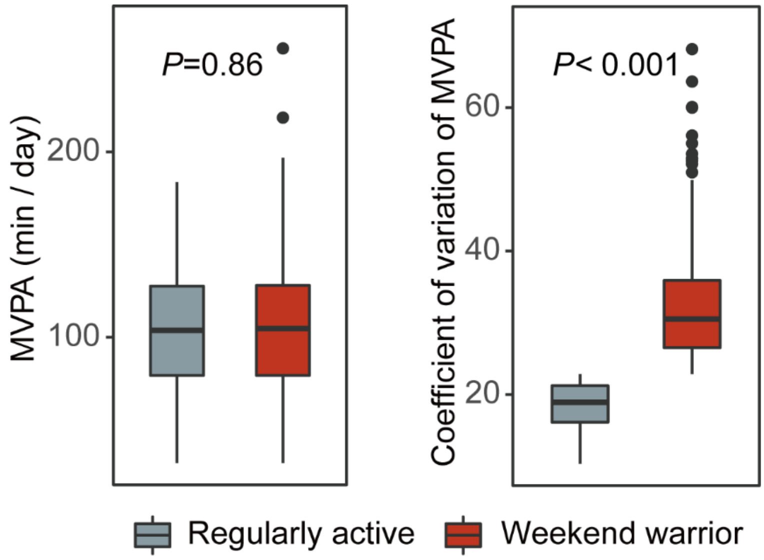

© 2024 Gou W et al. *JAMA Network Open.*
