# Supplementary material for: Physical Activity During Pregnancy and Preterm Birth Among Women With Gestational Diabetes
Source: JAMA Netw Open. 2024 Dec 19;7(12):e2451799. doi: 10.1001/jamanetworkopen.2024.51799 (PMC11659911; doi:10.1001/jamanetworkopen.2024.51799)
Supplement: Supplement 2. — Data Sharing Statement [file jamanetwopen-e2451799-s002.pdf]

Data Sharing Statement

Gou. Physical Activity During Pregnancy and Preterm Birth Among Women With Gestational Diabetes. *JAMA Netw Open*. Published December 19, 2024.
doi:10.1001/jamanetworkopen.2024.51799

Data
Data available: No

Additional Information
Explanation for why data not available: Data will be available upon request to the corresponding author.
